# Supplementary material for: Bridge ties bind collective memories
Source: Nat Commun. 2019 Apr 5;10:1578. doi: 10.1038/s41467-019-09452-y (PMC6451000; doi:10.1038/s41467-019-09452-y)
Supplement: Supplementary file 1 — Supplementary Information [file 41467_2019_9452_MOESM1_ESM.pdf]

# SUPPLEMENTARY METHODS

**Supplementary Methods |** Story used in study phase and critical items, taken from Anderson & Pichert (1978).

The two boys ran until they came to the driveway. "See, I told you today was good for skipping school," said Mark. "Mom is never home on Thursday," he added. The pair strolled across the finely landscaped yard. "I never knew your place was so big," said Pete. "Yeah, but it's nicer now than it used to be since Dad had the new stone siding put on and added the fireplace."

There were front and back doors and a side door which led to the garage which was empty except for three parked 10-speed bikes. They went in the side door, Mark explaining that it was always open in case his younger sister got home earlier than their mother.

Pete wanted to see the house so Mark started with the living room. It, like the rest of downstairs, was newly painted. Mark turned on the stereo, the noise of which worried Pete." Don't worry, the nearest house is a quarter of a mile away," Mark shouted. Pete felt more comfortable observing that no houses could be seen in any direction beyond the huge yard.

The dining room, with all the china and silver, was no place to play, so the boys moved into the kitchen. Mark said they wouldn't go to the basement because it had been damp ever since the new plumbing had been installed.

"This is where my Dad keeps his famous paintings," Mark said as they peered into the den. Mark bragged that he could get spending money whenever he needed it since he'd

discovered that his Dad kept a lot in the desk drawer.

There were three upstairs bedrooms. Mark showed Pete his mother's closet which was filled with furs and the locked box which held her jewels. As they got to his sister's room, Mark took the laptop from her desk and carried it to his room. Mark bragged that the bathroom in the hall was his since one had been added to his sister's room for her use. The big highlight in his room, though, was a leak in the ceiling where the old roof had finally rotted.

After the tour, Pete packed his things and set off. He rushed to get home, as he knew that his grandmother would be concerned if he didn't arrive on time.

**Scoring Units**

1. Mom not home (on Thursday.)
2. Garage empty except for (3-10 speed) bikes.
3. Side door unlocked (in case his sister gets home early).
4. There is a stereo
5. The nearest house is (1/4 mile) away/there are no nearby houses.
6. There is china.
7. There is silver.
8. (The den has) paintings.
9. (Dad keeps) cash in his desk.
10. (Mom has a box with) Jewels
11. Mom has fine furs.
12. There is a laptop.
13. The yard is finely landscaped.
14. The house is big.
15. There is new stone siding.
16. There is a new fireplace.
17. The downstairs area is newly painted.
18. The basement is damp.
19. The basement has new plumbing
20. There are three bedrooms
21. (The sister's room has) a new bathroom.
22. There is a hall bathroom/There are two bathrooms.
23. There is a leak in Marks ceiling
24. The roof has rotted.
25. The boys ran to the driveway.
26. The boys are skipping school.
27. One boy is named Mark.

28. One boy is named Pete.
29. Mark gives Pete a tour of the house.
30. The boys listen to the stereo/Pete's worried about the stereo's noise.

## SUPPLEMENTARY REFERENCES

1. Anderson RC, Pichert JW (1978) Recall of previously unrecallable information following a shift in perspective. *J Verbal Learn Verbal Behav* 17(1):1–12.
