# Supplementary material for: Bridge ties bind collective memories
Source: Nat Commun. 2019 Apr 5;10:1578. doi: 10.1038/s41467-019-09452-y (PMC6451000; doi:10.1038/s41467-019-09452-y)
Supplement: Supplementary file 3 — Reporting Summary [file 41467_2019_9452_MOESM3_ESM.pdf]

nature research

Corresponding author(s): Alin Coman, Ph.D.

Reporting Summary

Nature Research wishes to improve the reproducibility of the work that we publish. This form provides structure for consistency and transparency in reporting. For further information on Nature Research policies, see Authors & Referees and the Editorial Policy Checklist.

Statistical parameters

When statistical analyses are reported, confirm that the following items are present in the relevant location (e.g. figure legend, table legend, main text, or Methods section).

n/a	Confirmed	
☐	☒	The exact sample size (*n*) for each experimental group/condition, given as a discrete number and unit of measurement
☐	☒	An indication of whether measurements were taken from distinct samples or whether the same sample was measured repeatedly
☐	☒	The statistical test(s) used AND whether they are one- or two-sided<br>*Only common tests should be described solely by name; describe more complex techniques in the Methods section.*
☒	☐	A description of all covariates tested
☐	☒	A description of any assumptions or corrections, such as tests of normality and adjustment for multiple comparisons
☐	☒	A full description of the statistics including central tendency (e.g. means) or other basic estimates (e.g. regression coefficient) AND variation (e.g. standard deviation) or associated estimates of uncertainty (e.g. confidence intervals)
☐	☒	For null hypothesis testing, the test statistic (e.g. *F*, *t*, *r*) with confidence intervals, effect sizes, degrees of freedom and *P* value noted<br>*Give P values as exact values whenever suitable.*
☒	☐	For Bayesian analysis, information on the choice of priors and Markov chain Monte Carlo settings
☒	☐	For hierarchical and complex designs, identification of the appropriate level for tests and full reporting of outcomes
☐	☒	Estimates of effect sizes (e.g. Cohen's *d*, Pearson's *r*), indicating how they were calculated
☐	☒	Clearly defined error bars<br>*State explicitly what error bars represent (e.g. SD, SE, CI)*

Our web collection on statistics for biologists may be useful.

Software and code

Policy information about availability of computer code

Data collection	Software Platform for Human Interaction Experiments (SoPHIE) is the software platform we used to collect the date in the lab. See here (https://www.sophie.uni-osnabrueck.de/) for information about software functions and operation.
Data analysis	SPSS was used to analyze the data

For manuscripts utilizing custom algorithms or software that are central to the research but not yet described in published literature, software must be made available to editors/reviewers upon request. We strongly encourage code deposition in a community repository (e.g. GitHub). See the Nature Research guidelines for submitting code & software for further information.

Data

Policy information about availability of data

All manuscripts must include a data availability statement. This statement should provide the following information, where applicable:
- Accession codes, unique identifiers, or web links for publicly available datasets
- A list of figures that have associated raw data
- A description of any restrictions on data availability

All data associated with this study is available on an open data platform [https://osf.io/fxky4/?view_only=ededdaf259e74523a59c08e9716aa025]

Field-specific reporting

Please select the best fit for your research. If you are not sure, read the appropriate sections before making your selection.

☐ Life sciences ☒ Behavioural & social sciences ☐ Ecological, evolutionary & environmental sciences

For a reference copy of the document with all sections, see nature.com/authors/policies/ReportingSummary-flat.pdf

Behavioural & social sciences study design

All studies must disclose on these points even when the disclosure is negative.

Study description	We use experimental manipulations in lab-created networks to investigate how the temporal dynamics of conversations shape the formation of collective memories. We show that when individuals that bridge between clusters (i.e., bridge ties) communicate early on in a series of networked interactions, the network reaches higher mnemonic convergence compared to when individuals first interact within clusters (i.e., cluster ties). This effect, we show, is due to the tradeoffs between initial information diversity and accumulated overlap over time.
Research sample	A total of 192 students (123 female, mean age 21.83 years, SD = 3.97) affiliated with Princeton University took part in the study voluntarily for either research credit or compensation. We used a sample of Princeton undergraduate/graduate students for convenience, but we believe the results are generalizable to other populations.
Sampling strategy	The stopping rule for participant recruitment was established based on the effect size obtained in a previous study that used aggregate measures of collective memory in networks (10). A sample size of 12 networks (196 participants) was deemed adequate to obtain an effect size of Cohen'd=1 for the planned between-condition comparisons. The relatively small sample size needed to reveal an effect is due to the fact that the aggregation procedure involves averaging over all pairwise scores within each network (i.e., 120 scores per network), which drastically reduces the standard deviation and standard error in each condition. No participants were excluded from analyses.
Data collection	The data was collected in the lab and required participants to type their answers on a computer terminal. The research assistants who were in the room at the same time with the participants were blind to the study's hypotheses, but not to the condition.
Timing	Data collection occurred within the period of a semester, between September (2017) and January (2018)
Data exclusions	No data was excluded from analysis.
Non-participation	No participants dropped out of the study.
Randomization	Participants were randomly assigned to be part of one of two conditions. The research assistants/experimenters had no control over participants signing up for the study. They were assigned to a network community based on the chronological order in which they signed up for the study.

Reporting for specific materials, systems and methods

Materials & experimental systems

n/a	Involved in the study	
☒	☐	Unique biological materials
☒	☐	Antibodies
☒	☐	Eukaryotic cell lines
☒	☐	Palaeontology
☒	☐	Animals and other organisms
☐	☒	Human research participants

Methods

n/a	Involved in the study	
☒	☐	ChIP-seq
☒	☐	Flow cytometry
☒	☐	MRI-based neuroimaging

Human research participants

Policy information about studies involving human research participants

Population characteristics	See above
Recruitment	Participants were recruited using the participation pool at Princeton University. No self-selection bias issues are known in

Recruitment	Princeton University's research pool.
